# Supplementary material for: Identifying SARS-CoV-2 antiviral compounds by screening for small molecule inhibitors of nsp12/7/8 RNA-dependent RNA polymerase
Source: Biochem J. 2021 Jul 2;478(13):2425–43. doi: 10.1042/BCJ20210200 (PMC8286815; doi:10.1042/BCJ20210200)
Supplement: Supplementary Figures S1-S6 and Tables S1-S4 [file BCJ-478-2425-s1.pdf]

Supplementary Figure legends

Supplementary Figure S1. (**A**) Schematic diagram illustrating the FRET-based primer extension assay. The RNA substrate is composed of a Cy3 fluorophore-containing template strand and an annealed primer strand. The substrate is incubated with RdRp, which extends the primer strand generating duplex RNA. After incubation, a quencher strand is added, which can only anneal to the template strand and quench Cy3 fluorescence if RNA synthesis did *not* occur. (**B**) Results of the FRET-based primer-extension assay shown in (A). The quencher strand was added after 60 min of RdRp and primed-substrate incubation and fluorescence was measured after another 35 min. (**C-E**) Strand displacement assay using the indicated concentrations Sf nsp12-F/7H8 in the absence (C) or presence of 0.5 mM Mn^{2+} (D) or 2 mM Mn^{2+} (E). (**F**) Strand displacement assay using Sf nsp12-F/7H8 in the presence (+NTPs) or absence (no NTP) of 300 μM NTPs.

Supplementary Figure S2. (**A**) Strand displacement assay using the indicated concentrations of insect cell (Sf)-expressed nsp12-HF/7/8. This experiment was performed in parallel with the experiment shown in Figure 1E.

Supplementary Figure S3. (**A**) Strand displacement assay using 150 nM Sf nsp12-HF/7L8 premixed with Sf 7H8 (1:5 ratio) in the presence (+) or absence (-) of 5% DMSO.

Supplementary Figure S4. High-throughput SARS-CoV-2 RdRp inhibitor screen. (**A**) Representative kinetic data from control reactions in the HTS screen. For screen analysis, reaction velocities were extracted from the slope. (**B**) Z-score distribution for the screen performed at 3.75 μM compound concentration. (**C-D**) Analysis of the screen by endpoint signal. Normalised endpoint signal is plotted against compound number for the screen performed at 1.25 μM (C) or 3.75 μM (D) compound concentration.

Supplementary Figure S5. Concentration-response curves for validation of selected compounds identified in SARS-CoV-2 RdRp screen. (**A**) The experiment was performed using 150 nM RdRp, 100 nM RNA substrate, 300 μM of each NTP and in the presence (+Triton) or absence (-Triton) of 0.01% Triton X-100. Fluorescence quenching properties of the compounds were tested to identify false positives that interfered with the fluorescent substrate (quenching). (**B**) Quenching controls for the experiment shown in Figure 5A. IC_{50} values were calculated using Prism software.

Supplementary Figure S6. Comparative dose–response curves of selected antiviral compounds against SARS-CoV-2 in cell culture. (A) Anti-SARS-CoV-2 activities of GSK-650394, C646, BH3I-1, MDK-83190 and Cefsulodin in combination with 0.5 μM remdesivir following protocol detailed in **Figure 6A**. Representative overlaid images showing N protein immunofluorescence (green) and DRAQ7 nuclei staining (red). **(B)** Dose-response curve analysis. Viral infection values represent the area of virus infected cells visualised by N protein staining (green curves) and, simultaneously, cell viability was measured as the area of

cells stained for DRAQ7 (red curves). Data is plotted as percentage normalised to 0.5 µM remdesivir only treated wells (100%). Values represent mean and standard deviation (SD) of 3 replicates. Areas were calculated using FIJI software and EC_{50} values were calculated using Prism software.

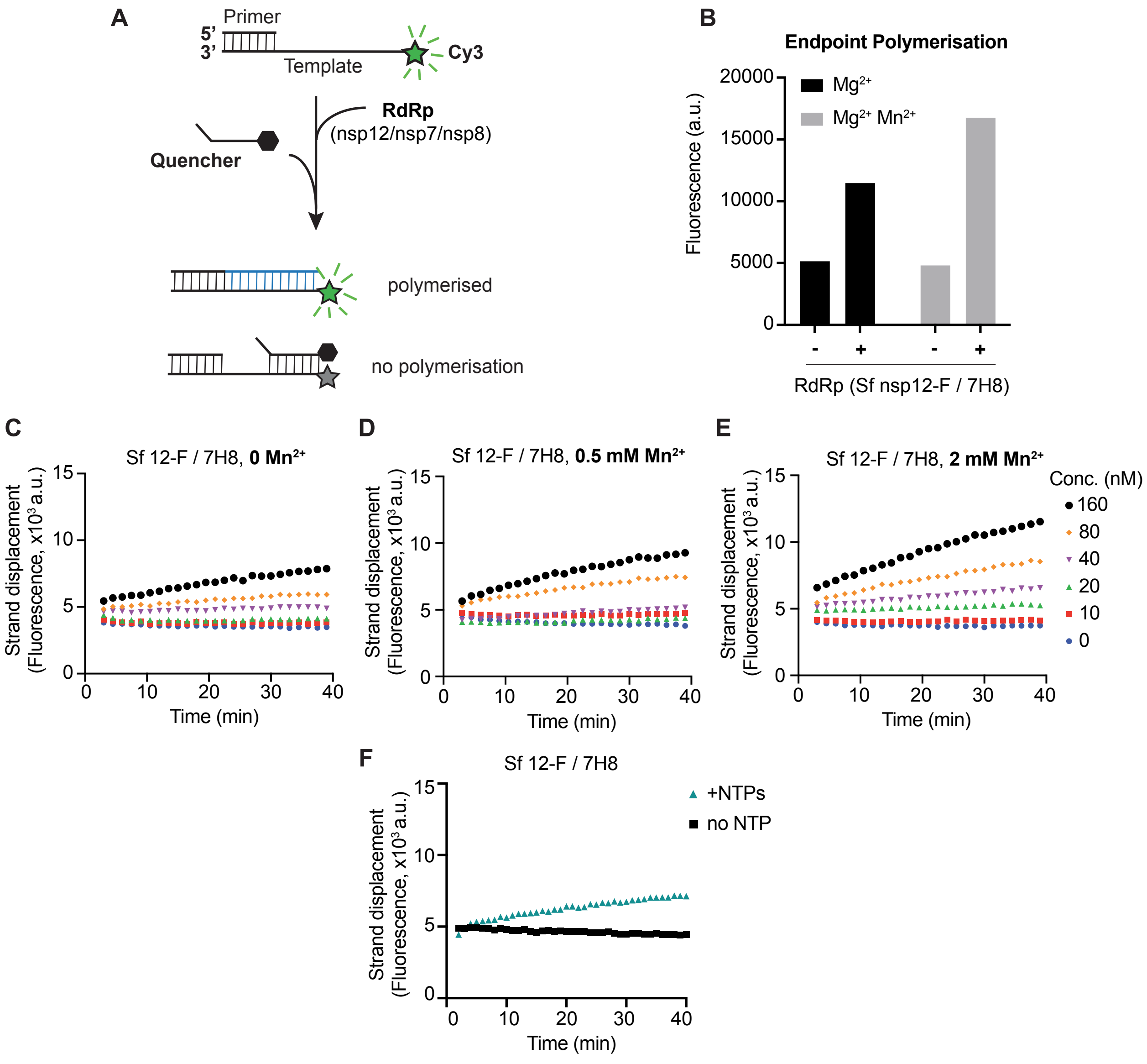

Supplementary Figure 1

A

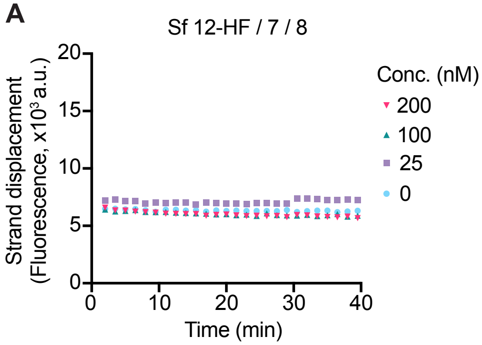

Supplementary Figure 2

A

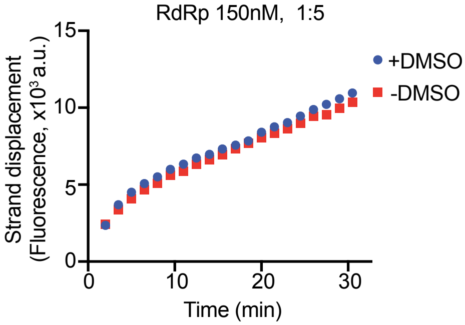

Supplementary Figure 3

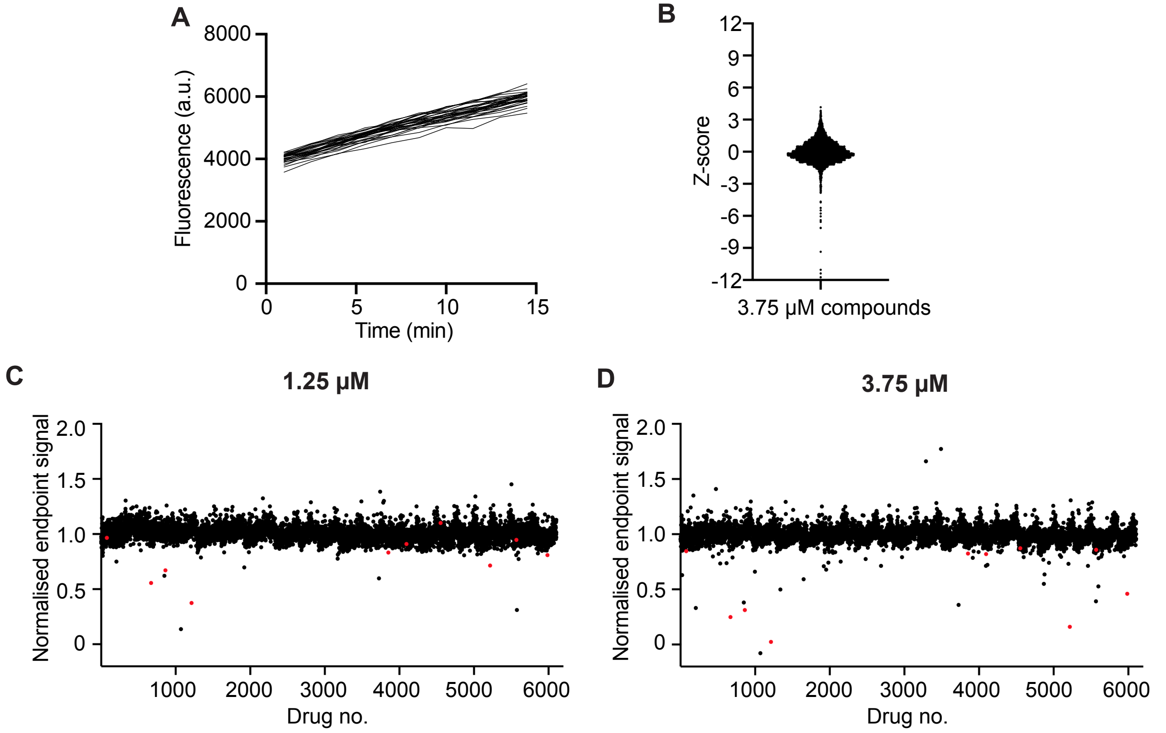

Supplementary Figure 4

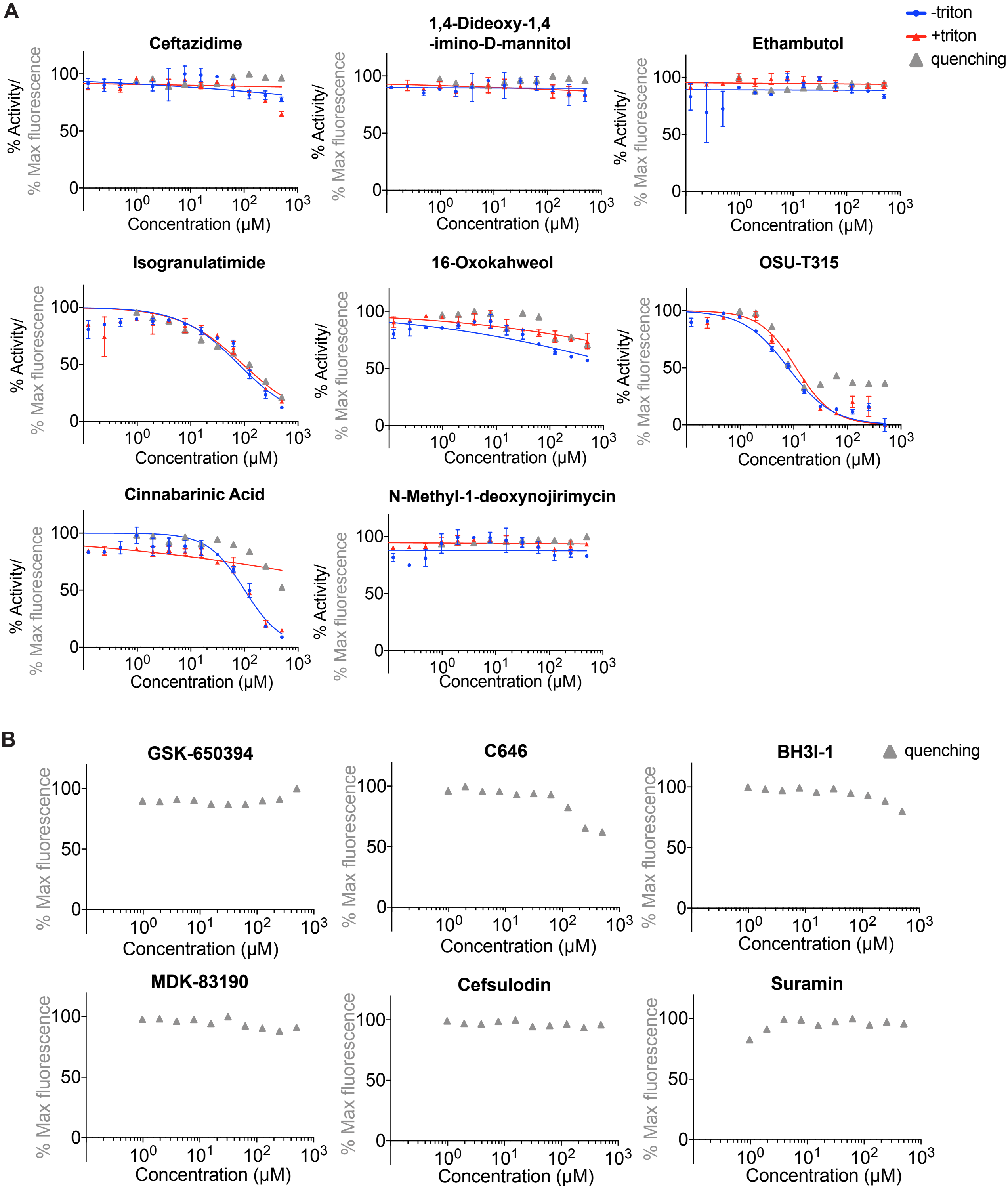

Supplementary Figure 5

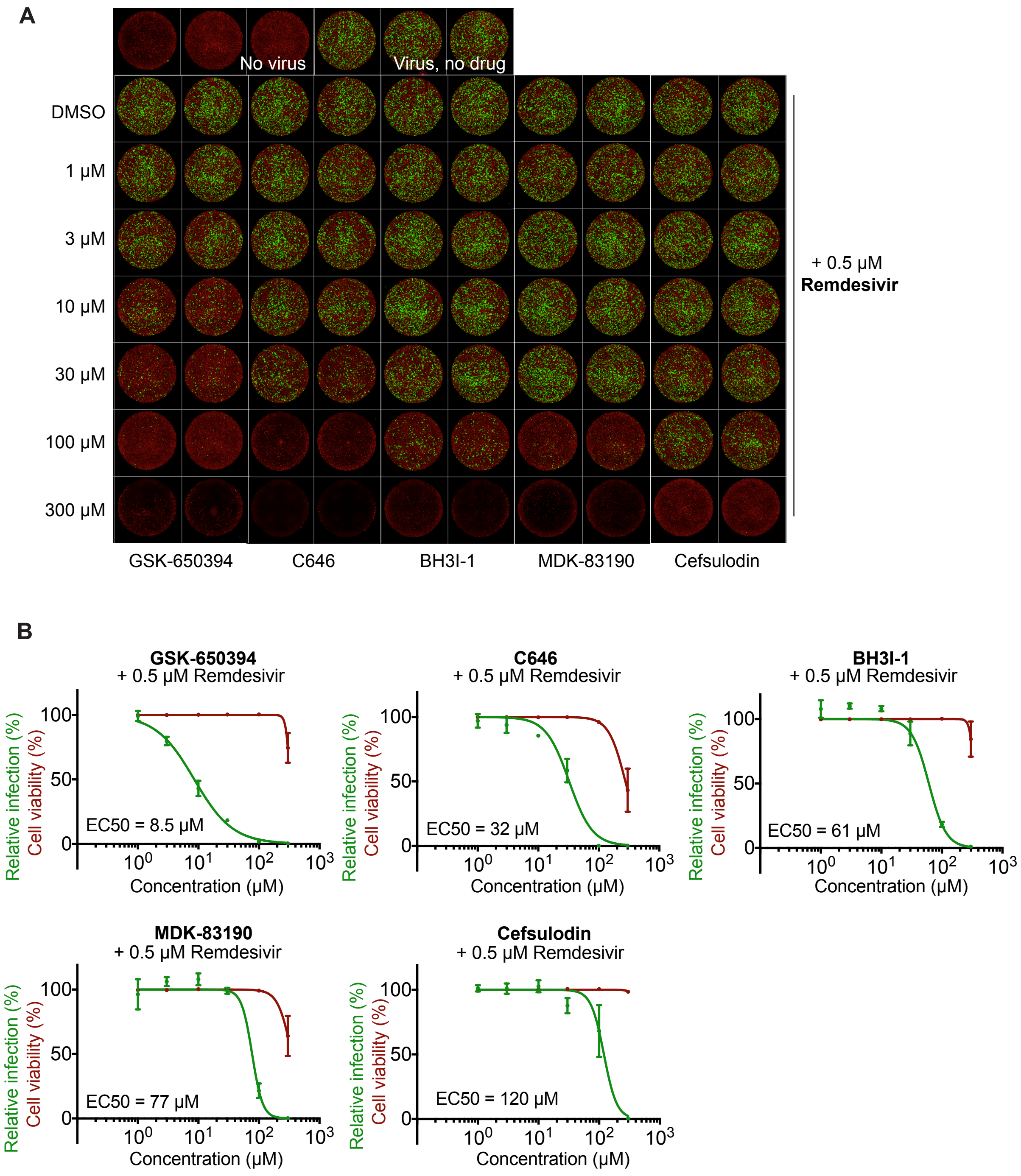

Supplementary Figure 6

Supplementary Table S1. Overview of purified SARS-CoV-2 RdRp proteins including yields

Protein preparation	Expression system	Yield (mg)	Yield (nmol)	Construct	Purification
**7H8**	Baculovirus-insect cell	19.5[a]	**607**[a]	nsp7-His6-nsp8	Ni-NTA, MonoQ, S200
**nsp12-HF**	Baculovirus-insect cell	1.92	17.3	nsp12-His6-3xFlag	Ni-NTA, MonoQ, S200
**nsp12-F/7H8**	Baculovirus-insect cell	0.084	0.59	nsp12-3xFlag/nsp7-His6-nsp8	FLAG M2, Heparin, S200
**nsp12-HF/7L8**	Baculovirus-insect cell	17.0[a]	**119**[a]	nsp12-His6-3xFlag/nsp7-GGSGGS-nsp8	Ni-NTA, MonoQ, S200
**nsp12-HF/7/8**	Baculovirus-insect cell	0.053	0.37	nsp12-His6-3xFlag/nsp7/nsp8	FLAG M2, Heparin, S200
**nsp7**	*E. coli*	1.96	213	14His-Sumo-nsp7	Ni-NTA, Ulp1, Ni-NTA, MonoQ, S200
**nsp8**	*E. coli*	5.34	244	14His-Sumo-nsp8	Ni-NTA, Ulp1, Ni-NTA, MonoQ, S200
**nsp12**	*E. coli*	0.65	6.1	14His-Sumo-nsp12	Ni-NTA, Ulp1, Ni-NTA, MonoQ, S200

[a] Combined yield from 2 preparations.

Supplementary Table S2. Predicted aggregation propensity of 18 HTS hit compounds

Compound name	LogP	Structural Similarity Index
NF 023	-6.1	-
Suramin	-5.7	-
Cefsulodin	-5.7	-
Ceftazidime	-5	-
PPNDS	-3.3	-
Evans Blue	-2.7	-
Diphenyl Blue	-2.7	-
1,4-Dideoxy-1,4-imino-D-mannitol	-2.6	-
N-Methyl-1-deoxynojirimycin	-2.2	-
Ethambutol	0.4	-
Cinnabarinic Acid	1.1	-
Isogranulatimide	2	-
BH3I-1	3.1	-
16-Oxokahweol	3.3	-
MDK 83190	3.8	-
C646	4.7	-
OSU-T315	5.2	-
GSK-650394	5.8	-

Compounds analysed using the open access tool Aggregator Advisor
See (Irwin et al., 2015)

Supplementary Table S3. Compounds that showed only weak or no clear activity against SARS-CoV-2 RdRp *in vitro* or interfered with the fluorescent substrate by quenching

Compound name	IC_{50} (µM) No detergent	IC_{50} (µM) + 0.01% Triton X-100	Data figure
**Cinnabarinic acid**	>100	>100	S4A
**Ceftazidim**	no clear inhibition	no clear inhibition	S4A
**1,4-Dideoxy-1,4-imino-D-mannitol**	no clear inhibition	no clear inhibition	S4A
**Ethambutol**	no clear inhibition	no clear inhibition	S4A
**16-Oxokahweol**	no clear inhibition	no clear inhibition	S4A
**N-Methyl-1-deoxynojirimycin**	no clear inhibition	no clear inhibition	S4A
**OSU-T315**	[a]	[a]	S4A
**Isogranulatimide**	[a]	[a]	S4A

[a] IC_{50} could not be assessed due to compound-related quenching effects on fluorescent substrate

Supplementary Table S4. Purchased drugs for *in vitro* validation and viral inhibition experiments

Compound name	CAS Number	Company	Catalog Number
**Suramin**	129-46-4	Sigma	S2671
**Cefsulodin**	52152-93-9	Sigma	C 8145
**Cinnabarinic Acid**	606-59-7	Sigma	SML0096
**1,4-Dideoxy-1,4-imino-D-mannitol**	114976-76-0	Sigma	D 8390
**C646**	328968-36-1	Sigma	SML0002
**OSU-T315**	2070015-22-2	MedChem Express	HY-18676
**MDK 83190**	79183-19-0	MedChem Express	HY-18633
**Ceftazidime**	72558-82-8	MedChem Express	HY-B0593
**BH3I-1**	300817-68-9	MedChem Express	HY-100383
**Remdesivir**	1809249-37-3	MedChem Express	HY-104077
**Isogranulatimide**	244148-46-7	Calbiochem/Millipore	371957
**16-Oxokahweol**	108664-99-9	Cayman Chemicals	CAY30117
**N-Methyl-1-deoxynojirimycin**	69567-10-8	Biovitica	BVT-0130-M001
**Ethambutol**	1070-11-7	Selleck Chemicals	S4004
**GSK-650394**	890842-28-1	Tocris	3572
